# Antinociceptive Activity of *Stephanolepis* *hispidus* Skin Aqueous Extract Depends Partly on Opioid System Activation

**DOI:** 10.3390/md11041221

**Published:** 2013-04-10

**Authors:** Vinicius Carvalho, Lohengrin Fernandes, Taline Conde, Helena Zamith, Ronald Silva, Andrea Surrage, Valber Frutuoso, Hugo Castro-Faria-Neto, Fabio Amendoeira

**Affiliations:** 1 Laboratory of Inflammation, IOC, Fiocruz, Rio de Janeiro-RJ, 21045-900, Brazil; 2 Division of Marine Ecosystems, Department of Oceanography, IEAPM, Arraial do Cabo-RJ, 28930-000, Brazil; E-Mail: lohengrin.fernandes@gmail.com; 3 Laboratory of Pharmacology, INCQS, Fiocruz, Rio de Janeiro-RJ, 21045-900, Brazil; E-Mails: taline.conde@incqs.fiocruz.br (T.C.); helena.zamith@incqs.fiocruz.br (H.Z.); ronald.silva@incqs.fiocruz.br (R.S.); fabio.amendoeira@incqs.fiocruz.br (F.A.); 4 Laboratory of Immunopharmacology, IOC, Fiocruz, Rio de Janeiro-RJ, 21045-900, Brazil; E-Mails: andrea.surrage@gmail.com (A.S.); frutuoso@ioc.fiocruz.br (V.F.); hcastro@ioc.fiocruz.br (H.C.-F.-N.)

**Keywords:** *Stephanolepis hispidus*, fish skin, antinociceptive, peixe-porco, marine products

## Abstract

*Stephanolepis hispidus* is one of the most common filefish species in Brazil. Its skin is traditionally used as a complementary treatment for inflammatory disorders. However, there are very few studies on chemical and pharmacological properties using the skin of this fish. This study was undertaken in order to investigate the effect of aqueous crude extract of *S. hispidus* skin (SAE) in different nociception models. Here, we report that intraperitoneal administration of SAE inhibited the abdominal constrictions induced by acetic acid in mice. In addition to the effect seen in the abdominal constriction model, SAE was also able to inhibit the hyperalgesia induced by carrageenan and prostaglandin E2 (PGE2) in mice. This potent antinociceptive effect was observed in the hot plate model too, but not in tail-flick test. Naloxone, an opioid receptor antagonist, was able to block the antinociceptive effect of SAE in the abdominal constriction and hot plate models. In addition, SAE did not present cytotoxic or genotoxic effect in human peripheral blood cells. Our results suggest that aqueous crude extract from *S. hispidus* skin has antinociceptive activity in close relationship with the partial activation of opioid receptors in the nervous system. Moreover, aqueous crude extract from *S. hispidus* skin does not present toxicity and is therefore endowed with the potential for pharmacological control of pain.

## 1. Introduction

Ninety percent of the world living biomass is in the oceans and this is equivalent to half of global biodiversity. The marine environment is an extraordinary reservoir of bioactive natural products which exhibit chemical and structural characteristics that are not found in natural products from terrestrial species [1,2]. Although the oceans are a rich source of bioactive compounds, the interest from pharmaceutical companies and research institutions in these natural products started only approximately 50 years ago. Since then, several drugs have been developed from marine products, including cytarabine (Cytosar-U^®^, Depocyt^®^) and trabectedin (Yondelis^®^) for the treatment of cancer; vidarabine (Vira-A^®^) for the treatment of virus infection; ziconotide (Prialt^®^) for the treatment of moderate to severe pain. Moreover, more than 14 thousand different marine natural products have been described and several of these are currently in pre-clinical and clinical evaluation, especially for the treatment of cancer, inflammatory diseases and pain [3,4,5,6,7]. 

The family Monacanthidae includes about 102 species in 32 genera of marine filefishes distributed worldwide [8]. Most species inhabit tropical and subtropical shallow waters (up to 300 m), usually associated with rocky shores, coral reefs, and muddy and sand bottoms, where they feed on a wide variety of benthic organisms, as sponges and algae [8,9]. Until now, four species of monacanthid have been registered in the Southwestern Atlantic Ocean—*Aluterus schoepfii*, *Aluterus monoceros*, *Monacanthus ciliatus* and *Stephanolepis* *hispidus* [10,11,12]. *S.* *hispidus* is one of the most common filefish species in Brazil and is popularly known as “peixe-porco”, “peroá” or “cangulo”. Although some people living in the northern coast of Rio de Janeiro, Southeast of Brazil, usually consume *S.* *hispidus* and others filefishes as the main source of protein, meanwhile the *S. hispidus* skin is discarded [13]. Many traditional fishermen in those areas consume water infusion of dried and powdered skin of filefishes as a complementary treatment for inflammatory disorders of the respiratory system [14]. Moreover, the aqueous extract of *S. hispidus* skin reduced the blood pressure of l-NAME-induced hypertension rats [15]. 

Mediators produced at the sites of inflammation have been known to produce pain through activation or sensitization of nociceptors adjacent to the injured tissue. Experimental models of inflammatory pain in rodents have been successfully employed to reproduce this kind of pain. These experimental models are used to search new anti-inflammatory and analgesic drugs [16]. Considering the potential use of marine products in the development of new drugs, the present study was carried out in order to analyze the effect of aqueous crude extract of *S. hispidus* skin in different nociception models.

## 2. Results and Discussion

The acetic acid-induced writhing model is normally used in screening the antinociceptive effects of drugs. This is an interesting model to study pain, because it has a good correlation between the analgesic effect obtained in animals and in humans [17]. Acetic acid induces local production of inflammatory mediators, including prostaglandins, which will sensitize the nociceptors leading to hyperalgesia. These nociceptive neurons are sensitive to nonsteroidal anti-inﬂammatory drugs (NSAIDs) and opioids [18]. In the present study, we aimed to evaluate the antinociceptive effect of aqueous crude extract of *S. hispidus* skin (SAE).

**Figure 1:**
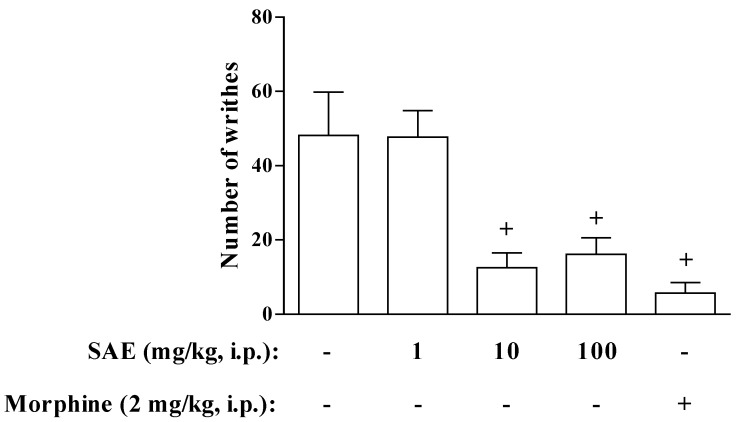
Dose-Response curve for the antinociceptive effect of *S. hispidus* skin (SAE) on acetic acid-induced writhes in mice. The number of writhes was counted over a 10-min period following intraperitoneally (i.p.) injection of acetic acid (0.8%). SAE and morphine were administered 1 h before acetic acid injection. Each bar is the mean ± SEM of six animals. ^+^ *p* < 0.05 when compared to untreated group.

The intraperitoneally (i.p.) injection of SAE, 1 h before acetic acid administration, potently inhibited the writhing reaction at doses of 10 and 100 mg/kg. However, i.p. injection of 1 mg/kg of SAE was not able to inhibit the acetic acid-induced abdominal writhing. As a control, we showed that morphine also inhibited the number of writhes (Figure 1). Thus, even a crude extract of this Monacanthidae skin had potent antinociceptive activity. However, in contrast with the reports by Mucillo-Baisch *et al.* [15], we did not observe the effect when the extract was administered by oral route, even with dose of 1000 mg/kg (Table 1), indicating that the analgesic effect of SAE is probably not mediated by nitrite, and that nitrate is present in the skin of *S. hispidus*. This absence of antinociceptive effect of SAE when it was given by gavage could be explained by the pharmacokinetics characteristics of SAE compounds. For instance, the active ingredient of SAE may suffer from first-pass metabolism in the liver, as observed in several drugs including morphine, whose oral bioavailability is only about 25% [19]. Another possibility is that the active component present in the SAE has a protein profile. The lack of measurable serum concentrations of proteic drugs, including Interferon, following oral administration was not unexpected since degradation of proteins in the gastrointestinal tract is a natural process of digestion [20]. 

**Table 1:** SAE does not inhibit acetic acid-induced writhes in mice when administered by gavage.

Treatment	(mg/kg)	Number of writhes
SAE	-	40 ± 4
	10	40 ± 8
	100	35 ± 3
	1000	30 ± 4

The number of writhes was counted over a 10-min period following i.p. injection of acetic acid (0.8%). SAE was administered 1 h before acetic acid injection. Each bar is the mean + SEM of six animals.

**Figure 2:**
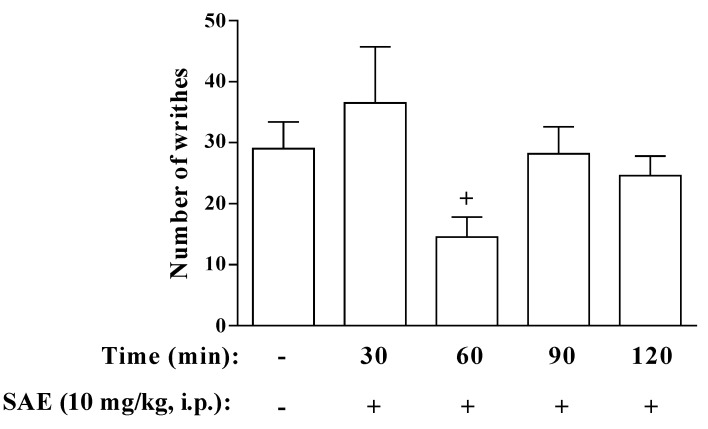
Time-Response curve for the antinociceptive effect of SAE on acetic acid-induced writhes in mice. The number of writhes was counted over a 10-min period following i.p. acetic acid injection (0.8%). SAE was administered at different intervals of time before acetic acid injection. Each bar is the mean ± SEM of six animals. ^+^ *p* < 0.05 when compared to untreated group.

The time-course of the antinociceptive effect of SAE (Figure 2) was evaluated from 30 to 120 min before acetic acid injection. We showed that SAE was able to induce analgesia only when it was given 60 min before acetic acid. To confirm that the analgesic effect of SAE is not associated with endotoxin contamination, we evaluated the endotoxin content in the SAE by Limulus Amebocyte Lysate (LAL) assay. We noted that SAE presents a very little contamination with endotoxin (11 ± 0.04 ng endotoxin/mg extract, median ± SEM, *n* = 4). Furthermore, Lipopolysaccharide (LPS) (110 ng/kg, i.p.) did not alter the acetic acid-induced writhing (from 26 ± 2.2 to 19 ± 3.1 number of writhes, median ± SEM, *n* = 5, saline and LPS respectively). These results indicated that the antinociceptive effect of SAE was not associated with endotoxin contamination, since when we injected the same dose of endotoxin present in 10 mg/kg of SAE we did not observe the same antinociceptive effect.

**Figure 3:**
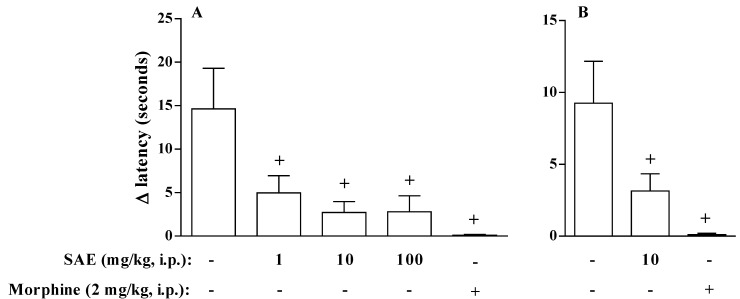
SAE inhibits inflammation-induced hyperalgesia in rats. SAE and morphine were administered 1 h before (**A**) carrageenan (250 μg/paw) or (**B**) PGE2 (1 μg/paw) injection. 15 min after intraplantar inflammatory stimuli, animals were placed on a hot plate at 54 ± 1 °C. Results show the latency time for hind paws. Each bar is the mean ± SEM of six animals. ^+^ *p* < 0.05 when compared to untreated group.

The antinociceptive effect of SAE was confirmed using the carrageenan- or PGE_2_-induced hyperalgesia. We observed that the i.p. injection of SAE 1 h before the stimulation with carrageenan inhibited hyperalgesia in all doses tested. As a control, we showed that morphine also inhibited the hyperalgesia-induced by carrageenan (Figure 3). Hyperalgesia is frequently detected in association with inflammation and is referred to as “inflammatory pain” [21]. This phenomenon is the result of the release of several inflammatory mediators, including PGE_2_, which act to increase the sensibility of the nociceptor fibers [22,23,24]. Considering the fact that SAE has also the ability to inhibit hyperalgesia induced by carrageenan, we analyzed the effect of SAE on hyperalgesia induced by an isolated inflammatory mediator involved in this phenomenon. We showed that SAE (10 mg/kg, i.p.) also inhibited PGE_2_-induced hyperalgesia in rats (Figure 3), suggesting that SAE could act as an antagonist of PGE_2_ receptor or have an activity in neurogenic nociception.

**Figure 4:**
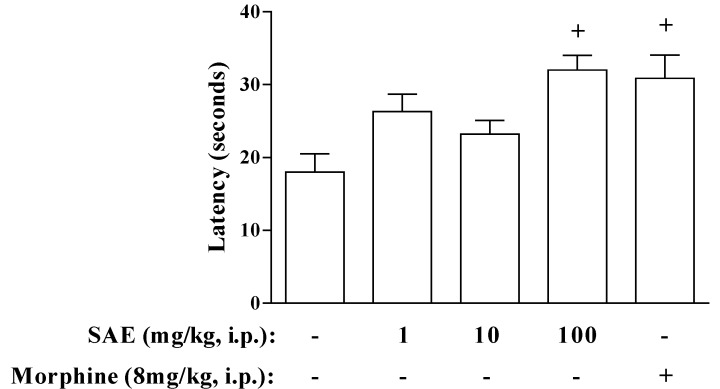
SAE inhibits reaction time evoked by thermal stimuli (hot plate) in mice. SAE was administered 1 h before placing the animals on hot plate at 54 ± 1 °C. Morphine was administered 30 min before placing the animals on hot plate at 54 ± 1 °C. Each bar is the mean ± SEM of six animals. ^+^ *p* < 0.05 when compared to untreated group.

To confirm the hypothesis that SAE could be acting in neurogenic nociception, we used the hot plate and tail-flick tests. In the hot plate model, SAE presented antinociceptive effect only at the higher dose (100 mg/kg, i.p.) (Figure 4), suggesting that the SAE could be acting through neurogenic mechanisms of pain. As a control, we showed that morphine also induced an antinociceptive effect in this model (Figure 4). However, SAE (100 mg/kg, i.p.) did not alter the tail-flick reflex (from 8 ± 0.8 to 7 ± 0.9 s, median ± SEM, *n* = 5, saline and SAE respectively) while morphine was able to induce the antinociceptive effect in this model (20 ± 1.8 s, median ± SEM, *n* = 5). Further information that we can infer with these data is that SAE probably acts in an opioid system through a direct action on supraspinal opioid receptors and not on spinal structures, because the tail flick test is appropriate for the detection of antinociception that is mediated predominantly by spinal mechanisms, whereas the hot plate test is appropriate for the detection of antinociception that is mediated predominantly by supraspinal structures [25,26].

**Figure 5:**
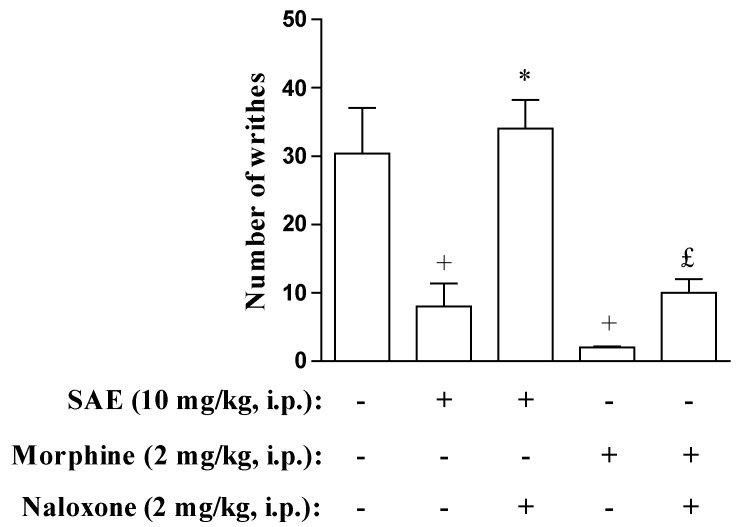
Naloxone inhibits antinociceptive effect of SAE in acetic acid-induced writhes. The number of writhes was counted over a 10 min period following i.p. acetic acid injection (0.8%). Naloxone was injected 30 min before SAE or morphine administration. SAE and morphine were administered 1 h before acetic acid injection. Each bar is the mean ± SEM of six animals. ^+^ *p* < 0.05 when compared to untreated group. * *p* < 0.05 when compared to SAE-treated group. ^£^ *p* < 0.05 when compared to morphine-treated group.

**Figure 6:**
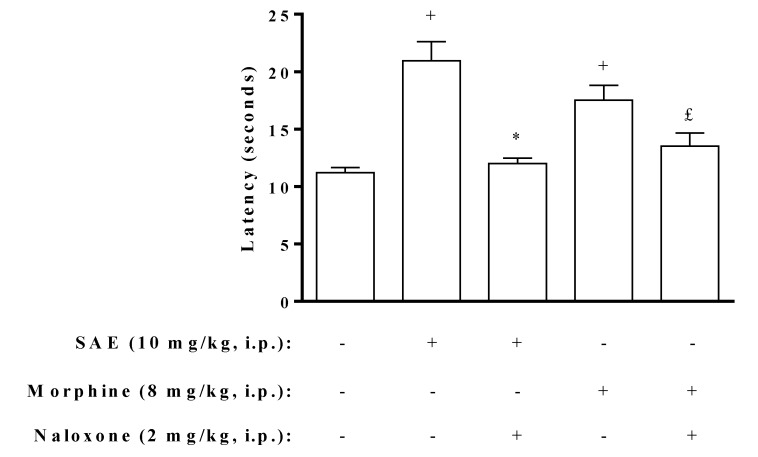
Naloxone inhibits antinociceptive effect of SAE in thermal stimuli (hot plate) in mice. Naloxone was injected 30 min before SAE or morphine administration. SAE was administered 1 h before placing the animals on hot plate at 54 ± 1 °C. Morphine was administered 30 min before placing the animals on hot plate at 54 ± 1 °C. Each bar is the mean ± SEM of six animals. ^+^ *p* < 0.05 when compared to untreated group. * *p* < 0.05 when compared to SAE-treated group. ^£^ *p* < 0.05 when compared to morphine-treated group.

For the assessment of opioid system involvement in the antinociceptive activity of SAE, animals were pre-treated with an opioid receptor antagonist, naloxone. We showed that naloxone abolished the antinociceptive effect of SAE and morphine in the abdominal constriction and hot plate models (Figure 5, Figure 6), suggesting that at least part of the antinociceptive effect observed for the extract is due to the involvement of opioid system. Nevertheless, since SAE is an unpurified extract one must keep in mind that different bioactive substances may be exerting the antinociceptive effect attributed to SAE. New experiments using purified fractions of SAE would be necessary to clarify this point. 

The evaluation of genotoxicity and cytotoxicity is crucial to the development of new drugs, since the absence of the genotoxic and cytotoxic effects improve the potential safety of these compounds [27]. As shown in Table 2, SAE did not present genotoxic neither cytotoxic effect in a range of concentrations between 0.05 and 5000 μg/mL in human blood cells. In genotoxicity assay, most of the cells examined on slides were undamaged, few cells showed minor damage (class 1) and very few showed a large amount of damage (classes 2 and 3). Furthermore, there were no significant differences in DNA damage between the different SAE concentrations tested. As expected, methyl methane-sulfonate (MMS), a positive control, induced a significant increase (*p* < 0.01) in DNA fragmentation in peripheral blood cells. Thus, these data suggest neither genotoxicity (*i.e.*, no increase in DNA damage) nor cytotoxicity for SAE.

**Table 2:** Detection of cytotoxicity and DNA damage in human peripheral blood after *in vitro* treatment with different concentrations of SAE.

Treatments (μg/mL)		Levels of DNA damage (%)				DNA damage score Total arbitrary units	Cytotoxicity (% of death cells)
		0	1	2	3		
SAE	-	94.00 ± 2.00	5.00 ± 1.50	0.50 ± 0.00	0.50 ± 0.50	15.00 ± 6.00	0.00
	0.05	97.00 ± 0.00	2.75 ± 0.25	0.25 ± 0.25	0.00 ± 0.00	6.50 ± 0.50	0.00
	0.5	97.00 ± 0.50	2.25 ± 0.75	0.25 ± 0.25	0.50 ± 0.00	8.50 ± 0.50	0.00
	5	95.75 ± 0.25	3.75 ± 0.75	0.25 ± 0.25	0.25 ± 0.25	10.00 ± 1.00	0.00
	50	95.75 ± 0.75	4.00 ± 1.00	0.25 ± 0.25	0.00 ± 0.00	9.00 ± 1.00	0.00
	500	94.00 ± 2.50	6.00 ± 2.50	0.00 ± 0.00	0.00 ± 0.00	12.00 ± 5.00	0.00
	1000	93.25 ± 3.25	6.50 ± 3.00	0.00 ± 0.00	0.25 ± 0.25	14.50 ± 7.50	0.50
	2500	92.50 ± 1.50	6.75 ± 1.75	0.75 ± 0.25	0.00 ± 000	16.50 ± 2.50	0.00
	5000	91.50 ± 3.00	8.50 ± 3.00	0.00 ± 0.00	0.00 ± 0.00	17.00 ± 6.00	0.50
MMS		0.00 ± 0.00	6.75 ± 6.75	7.75 ± 4.25	85.50 ± 11.0	557.50 ± 35.50 *	-

The results are expressed as mean ± SEM; MMS (methyl methane-sulfonate): Positive control. * *p* < 0.01 when compared to untreated group.

## 3. Experimental Section

### 3.1. Biological Materials

Samples of the ﬁsh *S.* *hispidus* were collected in the coastal region of Sepetiba Bay (23°00′00″S/43°55′00″W), Rio de Janeiro State, Brazil in February 2009. The specimen was identified by Magda Fernandes from the Department of Marine Biology, Laboratory of Living Resources of the Universidade Federal do Rio de Janeiro (UFRJ). The animals were frozen at −20 °C until its use in the assays. Voucher specimens of *S. hispidus* were permanently stored at Instituto de Estudos do Mar Almirante Paulo Moreira collection (IEAPM-DRMV1087), Arraial do Cabo, Rio de Janeiro, Brazil.

### 3.2. Preparation of Extract

Fish skins were dried at room temperature and grinded. The aqueous crude extract of *S.* *hispidus* skin (SAE) were obtained by infusion with 1 L of boiling distilled water of triturated skin (250 g), followed by filtration and lyophilization. At the time of use, extract was reconstituted in 0.9% sterile saline at the required doses.

### 3.3. Animals

Male Swiss-Webster mice (20–30 g) and Wistar rats (180–200 g) were obtained from the Oswaldo Cruz Foundation breeding colony and the experiments in this study received prior approval from the Committee on Use of Laboratory Animals of the Oswaldo Cruz Foundation (CEUA-FIOCRUZ, license L-010/05). The animals were maintained with free access to food and water and kept at 25–28 °C under a controlled 12 h light/dark cycle. Experiments were performed during the light phase of the cycle. The animals were allowed to adapt to the laboratory for at least 2 h before testing and used only once.

### 3.4. Abdominal Writhing Test

The methods described by Koster *et al.* [28] were used with few modifications. In brief, the selected groups of animals, consisting of six mice per dose of fish skin extract, LPS or vehicle, were used in the test. Animals were pre-treated with different doses of SAE, i.p. or per oral route (p.o.), at different intervals of time prior to the i.p. injection of acetic acid 0.8%. In some cases, LPS was administered i.p. 1 h before the injection of acetic acid. Five minutes after the i.p. injection of acetic acid, the number of writhes exhibited by each mouse was counted for 10 min. Control mice received sterile saline as vehicle or morphine (2 mg/kg). In some experiments, naloxone (2 mg/kg body weight, i.p.), an opioid receptor antagonist, was administered 30 min before SAE. 

### 3.5. Hot Plate Test

The hot-plate test was used to measure reaction times according to the method described by Amendoeira *et al.* [29]. Mice were placed individually on a hot-plate metallic surface (model DS37, Ugo Basile, Varese, Italy) maintained at 54 ± 1 °C, and the time between placement of the animal on the hot-plate and the occurrence of either the licking of the hind paws, shaking or jumping off from the surface was recorded as reaction time (s). Reaction time was measured at 1 h following treatment with different doses of SAE or vehicle, with a cut-off time of 30 s. Different doses of SAE were injected i.p. 1 h before mice were placed on hot plate. Control mice received i.p. sterile saline as vehicle or morphine (8 mg/kg).

### 3.6. Hyperalgesia

Hyperalgesia was induced by the intraplantar injection of carrageenan or PGE_2_ (Sigma Chemical Co, St. Louis, MO, USA) in the rat left hind paw. The hyperalgesia was evaluated based on method described by Lavich *et al.* [30], based on the Hargreaves method of nociceptive induction. Briefly, Wistar rats were divided in groups of six animals each. Carrageenan (250 μg/50 μL/paw) or PGE_2_ (1 μg/50 μL/paw) were injected into the left hind paws, while vehicle was injected into the right hind paws. Different doses of SAE were injected i.p. 1 h before the stimulation with carrageenan or PGE_2_. After 15 min, rats were individually placed on a hot plate with a fixed temperature of 54 ± 1 °C. The withdrawal response latency of each hind paw was determined using to separate chronographs. Control mice received i.p. sterile saline as vehicle or morphine (2 mg/kg). Hyperalgesia to heat was defined as a decrease in withdrawal latency and calculated as follows: Δ paw withdrawal latency (s) = right paw withdrawal latency—left paw withdrawal latency. The animals were kept no longer than 30 s over the hot plate to avoid any tissue damage. 

### 3.7. Tail-Flick Test

The nociceptive response was evaluated by recording the latency to withdrawal of the tail in response to heat from a light beam focused on the ventral tail surface [31]. The tails of mice were exposed to a focused beam of light from a 45-W projection bulb. The heat intensity was set by adjusting the source voltage for the bulb to 45 V. When a withdrawal response occurred, the stimulus was terminated and the response latency was measured electronically. A cut-off time of 30 s was used to avoid tissue damage. Changes in withdrawal response latency induced by the treatments were evaluated after 60 min.

### 3.8. Endotoxin Evaluation

Limulus Amebocyte Lysate (LAL) was used to measure the content of endotoxin in. Endotoxin evaluation of endotoxin content in aqueous crude extract of *S. hispidus* skin (SAE) was carried out using a Limulus Amebocyte Lysate (LAL) kit (Lonza, Maryland, MD, USA) according to the manufacturer’s instructions. Color intensity was measured at 405 nm using a microplate reader, SpectraMax M5 (Molecular Devices, California, CA, USA).

### 3.9. Toxicological Tests

#### 3.9.1. Cytotoxicity

Different concentrations of SAE (0.05–5000 μg/mL) in 0.9% NaCl (solvent-control) were added to heparinized peripheral human blood obtained by venipuncture immediately before the assays. Two hours after the treatments at 37 °C, cell death was evaluated using the fluorochrome-mediated viability assay performed according to Hartmann & Speit [32]. After treatments, whole blood cells (50 μL) were stained with an equal volume of the solution of 30 μg/mL fluorescein diacetate plus 8 μg/mL ethidium bromide in phosphate-buffered saline (PBS). Samples (50 μL) were spread on a microscope slide and observed under a fluorescence microscope with an excitation filter of 420–490 nm (blue light). Viable cells are stained in green while dead cells exhibit their nucleus stained in orange. A total of 200 cells were analyzed for each treatment. Survival percentage was obtained.

#### 3.9.2. Comet Assay

The alkaline version of the comet assay was performed as described by Speit & Hartmann [33]. DNA damage in whole blood was evaluated at the same concentrations of SAE as indicated above. Methyl methane-sulfonate (160 μM MMS: Aldrich, Milwaukee, WI, USA) was used as a positive control. Two hours after the treatments in duplicate, a sample (5 μL) was mixed with 120 μL of 0.5% low-melting-temperature agarose (LMPA: Sigma, St Louis, MO, USA) in PBS and applied to microscope slides pre-coated with 1.5% normal melting-point agarose in PBS (Sigma, St Louis, MO, USA). Four slides per treatment were prepared (two slides per culture). The slides were covered with a coverslip and refrigerated for 5 min. After LMPA solidification, the slides were immersed in ice-cold alkaline lysing solution (2.5 M NaCl, 10 mM Tris, 100 mM Na_2_EDTA, 10% dimethyl sulfoxide and 1% Triton X-100, 1% *N*-lauroylsarcosine sodium salt, pH 10) for at least 1 h at 4–5 °C. Then, the slides were placed for 20 min in ice-cold alkaline electrophoresis solution (0.3 M NaOH, 1 mM EDTA, pH 13), followed by electrophoresis at 25 V:300 mA (0.8–1.5 V/cm) for 20 min. All of the above steps (preparation of slides, lysis and electrophoresis) were conducted without direct light exposure, to prevent additional DNA damage. After electrophoresis, the slides were neutralized by three washings for 5 min each in Tris buffer (0.4 M Tris, pH 7.5), drained, fixed in absolute ethanol for about 10 min and left at room temperature overnight to dry. The slides were stained with ethidium bromide (20 μg/mL). Two hundred cells per treatment (four slides, 50 cells each) were analyzed under a fluorescence microscope with a blue (420–490 nm) excitation filter and yellow (520 nm) emission filter [32].

DNA damage was quantified by visual scoring, and the cells were placed into four classes according to tail size, between class 0 (undamaged cells) and maximum comet length (class 3) [34]. The DNA damage was expressed as percentage of cells into four classes and as arbitrary units (AU) according to the formula: AU = (0 × *n*_0_) + (1 × *n*_1_) + (2 × *n*_2_) + (3 × *n*_3_), where *n* = the number of cells analyzed in each class. Thus, the total damage score in AU (TAU) for 200 comets ranged from 0 (200 undamaged cells) to 600 (all cells presenting damage class 3).

### 3.10. Statistical Analysis

The results were expressed as mean ± S.E.M. Statistical significance was analyzed by the one-way analysis of variance (ANOVA) followed by the Newman-Keuls Student test for multiple comparisons. *P* values of 0.05 or less were considered as indicative of significance. In the comet assay, differences between the mean values of TAU for each concentration of SAE in relation to solvent-control group from two independent experiments were tested by the Students one-tailed t-test. In addition the effects of SAE on the intercellular distribution of DNA damage were tested for significance using one-way ANOVA followed by a Dunnett’s multiple comparison test.

## 4. Conclusions

Taken together, our results reveal that aqueous extract of *S. hispidus* skin has a potent antinociceptive activity and low toxicity, supporting the ethnopharmacological use of crude aqueous extract by Brazilian fishermen. Moreover, the antinociceptive effect of extract is tightly associated with activation of opioid system in supraspinal structures. This extract or its isolated chemical components could represent a new resource for the development of new marine-based therapy useful in the control of pain.
